# International Medical Congress, Berlin, 1890

**Published:** 1890-03

**Authors:** Rudolf Virchow

**Affiliations:** President; Vice-President; Vice-President; Vice-President; Secretary-General


### INTERNATIONAL MEDICAL CONGRESS, BERLIN, 1890.

### INVITATION TO THE TENTH INTERNATIONAL MEDICAL CONGRESS.

In accordance with the decision of the Ninth Congress at Washington, the Tenth International Medical Congress will be held at Berlin, from the 4th to the 9th of August, 1890.

By the delegates of the German Medical Faculties and the chief Medical Societies of the German empire, the undersigned have been appointed members of the General Committee of Organization. A special Committee of Organization has also been appointed for each of the different sections, to arrange the scientific problems to be discussed at the meetings of the respective Sections. An International Medical and Scientific Exhibition will also be held by the Congress.

We have the honor to inform you of the above decisions, and at the same time cordially to invite your attendance at the Congress. We should esteem it a favor if you would kindly extend this invitation to your friends in medical circles, as way may offer.

DR. RUDOLF VIRCHOW, President.
DR. VON BERGMANN,
DR. LEYDEN,
DR. WALDEYER,
Vice-Presidents.
DR. LASSAR, Secretary-General.

All communications must be directed to the General Secretary, Berlin NW., Karlstrasse 19.

